# Evaluation of the initial 12 months of a routine cryptococcal antigen screening program in reduction of HIV-associated cryptococcal meningitis in Uganda

**DOI:** 10.1186/s12913-022-07624-z

**Published:** 2022-03-04

**Authors:** Kagimu Enock, Kiwanuka Julius, Bridget C. Griffith, Derrick Bary Abila, Morris K. Rutakingirwa, John Kasibante, Kiiza Tadeo Kandole, Richard Kwizera, Aggrey Semeere, David B. Meya

**Affiliations:** 1 School of Medicine, College of Health Sciences, Makerere University, Kampala, Uganda; 2 Infectious Diseases Institute, College of Health Sciences, Makerere University, Kampala, P.O Box 7072, Uganda; 3 Makerere University School of Public Health, Kampala, Uganda; 4 University of Minnesota, School of Public Health, Minneapolis, MN USA; 5 McGill University, Montreal, Canada

## Abstract

**Background:**

Asymptomatic Cryptococcal Antigenemia (CrAg) patients develop meningitis within a month of testing positive. Pre-emptive antifungal therapy can prevent progression to Cryptococcal meningitis (CM). In April 2016, a national CrAg screening program was initiated in 206 high-volume health facilities that provide antiretroviral therapy in Uganda. We report the evaluation of the CrAg screening cascade focusing on linkage to care, fluconazole therapy for 10 weeks and 6 months follow up, and ART initiation in a subset of facilities.

**Methods:**

We conducted a retrospective, cross-sectional survey of patients with CD4 < 100 at seven urban and seven rural facilities after 1 year of program implementation. We quantified the number of patients who transitioned through the steps of the CrAg screening cascade over six-months follow-up. We defined cascade completion as a pre-emptive fluconazole prescription for the first 10 weeks. We conducted semi-structured interviews with lab personnel and clinic staff to assess functionality of the CrAg screening program. Data was collected using REDCap.

**Results:**

We evaluated 359 patient records between April 2016 to March 2017; the majority (358/359, 99.7%) were from government owned health facilities and just over half (193/359, 53.8%) had a median baseline CD4 cell count of < 50 cell/μL. Overall, CrAg screening had been performed in 255/359 (71.0, 95% CI, 66.0–75.7) of patients’ records reviewed, with a higher proportion among urban facilities (170/209 (81.3, 95% CI, 75.4–86.4)) than rural facilities (85/150 (56.7, 95% CI, 48.3–64.7)). Among those who were CrAg screened, 56/255 (22.0, 95% CI, 17.0–27.5%) had cryptococcal antigenemia, of whom 47/56 (83.9, 95% CI, 71.7–92.4%) were initiated on pre-emptive therapy with fluconazole and 8/47 (17.0, 95% CI, 7.6–30.8%) of these were still receiving antifungal therapy at 6 months follow up. At least one CNS symptom was present in 70% (39/56) of those with antigenemia. In patients who had started ART, almost 40% initiated ART prior to CrAg screening. Inadequacy of equipment/supplies was reported by 15/26 (58%) of personnel as a program barrier, while 13/26 (50%) reported a need for training about CM and CrAg screening.

**Conclusion:**

There was a critical gap in the follow-up of patients after initiation on fluconazole therapy. ART had been initiated in almost 40% of patients prior to CrAg screening.. Higher antigenemia patients presenting with CNS symptoms could be related to late presentation. There is need to address these gaps after a more thorough evaluation.

## Background

*Cryptococcus neoformans* is the most common cause of meningitis among adults with Human Immunodeficiency Virus (HIV) in sub-Saharan Africa (SSA), and it is associated with approximately 20–25% of acquired immunodeficiency syndrome (AIDS)-related deaths in this region [1–4]. Despite the accelerated access to antiretroviral therapy (ART) in the last decade, mortality among patients with HIV-associated cryptococcal meningitis (CM) has remained relatively high [5–7], and SSA carries a disproportionate burden of new infections [4, 8, 9]. Among patients who complete standard antifungal therapy for CM, survival at 2 years from diagnosis is 69% [10], and this proportion is lower in settings where optimal treatment regimens are not available [11].

Infection with *C. neoformans* can be diagnosed using a cryptococcal antigen (CrAg) test in serum/plasma before the infection progresses and the patient develops meningitis. The current management strategy for CM includes regimens that are expensive and not always available, especially in resource-limited settings [10, 12, 13]. There is evidence demonstrating the cost effectiveness of CrAg screening and CM pre-emptive therapy in routine care for people living with HIV [12, 14–16].

The prevalence of asymptomatic cryptococcal antigenemia in people living with HIV in Uganda with CD4 ≤ 100 cells/μl is estimated between 5 and 10% [17]. Asymptomatic patients with a positive CrAg test in blood (antigenemia) will typically develop meningitis in approximately 3 weeks [18]. This provides a window of opportunity in which treatment with fluconazole as pre-emptive therapy can prevent progression to CM [19]. This fluconazole has been provided at a free cost from most of government ART care and screening facilities in Uganda.

In order to increase the capacity to detect CM before it progresses to meningitis in patients with HIV in Uganda, a CrAg screening program was initiated in 206 high-volume (≥1000 patients) government and private health facilities providing ART through the Ugandan Ministry of Health.

The CrAg screening program was initiated with the training of key members of the treatment teams from US Centers for Disease Control and Prevention (CDC)/U.S. Presidents Emergency Plan for AIDS Relief (PEPFAR) implementing partners. The key members were then tasked to initiate trainings at the facility level. CDC provided the cryptococcal antigen screening kits for use during the roll-out in the 206 chosen health facilities. Since the initiation of the program in April 2016, there has not been a formal evaluation of the operations and outputs of this program. We therefore sought to understand the current operation of the program through; 1) quantification of metrics along the CrAg screening cascade and 2) identification of operational challenges cited by the implementers at selected health facilities.

## Methods

### Study design

We conducted a cross sectional study with two parts: Part 1 was a retrospective review of patient records in an electronic medical record (EMR) systems (Open MRS) [20] or in the hard copy CD4 registers, and Part 2 was a series of interviews conducted with key health facility staff.

### Identification and initial contact with facilities

Using the initial CrAg kit distribution list from the Infectious Diseases Institute (IDI) directory, we obtained health facilities’ contact information and contacted the selected health facilities via telephone ahead of the planned visit/data collection date. During the call, study personnel explained the study and procedures (abstraction of data from the EMR, registers and charts) and asked about the availability of the laboratory personnel and ART in-charges and the best days/times to schedule a study visit. Study personnel called the health facility approximately 2 days before the planned visit to remind them of the upcoming visit and what to expect during the visit. This was another opportunity for the facility to ask questions about the study. After agreement on the day and time, two study team members then went to the health facilities, did formal introductions, and identified the contact person managing the EMR or CD4 registers.

### Study sites and setting

Our evaluation included 14 health facilities located in two urban districts in Central Uganda: Kampala (capital city of Uganda) and Wakiso, and three rural districts in South Western Uganda: Masaka, Rakai, and Ssembabule. The 14 health facilities (Six health center level three, three health center level four, three general referral hospitals, and two regional referral hospitals) were at different tiers of the health care system delivery as follows: (1) level III (providing outpatient services, maternity, general ward and laboratory to a population of about 20,000 people); (2) level IV (providing all services at level III and including a theatre and blood transfusion to about 100,000 people); (3) general hospitals (provide all services at level IV and includes x-ray to a population of 100,000-1,000,000 people); and (4) regional referral hospitals (provide all services of general hospitals and includes specialist services to a population of about 1,000,000-2,000,000 people). Health facilities were selected using a convivence sample of those that responded to our request to visit.

### Sample size calculation

Using unpublished sample data from 15 HIV programs implementing partners, we estimated the proportion of patients with CD4 count less than 100 cells/μL and screened for CrAg to be 19%. Using a 95% confidence level and an expected error margin of 5%, we expected to review 236 patient charts from each of the two regions, giving an overall sample size of 472 patient charts. We conducted 26 individual interviews out of the 28 planned, targeting laboratory personnel and in-charge of the ART clinic, two from each of the participating 14 selected sites. The two individual interviews could not be conducted due to concurrent events at one of the facility. With a total of 26 interviews, the number was sufficient to reach saturation of the major themes related to the barriers and facilitators for CrAg screening.

### Data collection

#### Part 1: retrospective review of patient records

We abstracted data from the OpenMRS® medical records system or the CD4 and CrAg registers. These registers are standard Uganda Ministry of Health (MoH) tools that are used for documentation and generation of routine performance reports on selected CD4, viral load, and CrAg screening indicators from point of diagnosis to point of treatment or pre-emptive fluconazole (CrAg screening cascade). We operationally defined a successful cryptococcal care cascade when: at least 90% of patients with a CD4 ≤ 100 get a CrAg test, 90% of CrAg positive patients start fluconazole, and 90% of these complete 6 months of fluconazole [21]. We obtained clinic file numbers for every patient with a documented CD4 count ≤100 cells/μL, and we used the corresponding CrAg register and patient chart for each patient to determine the number that progressed through each level of the cryptococcal care cascade. The criteria for inclusion in the study was: patients with HIV that had a recorded CD4 cell count of ≤100 cells/μL between April 2016 to March 2017.

**Fig. 1:**
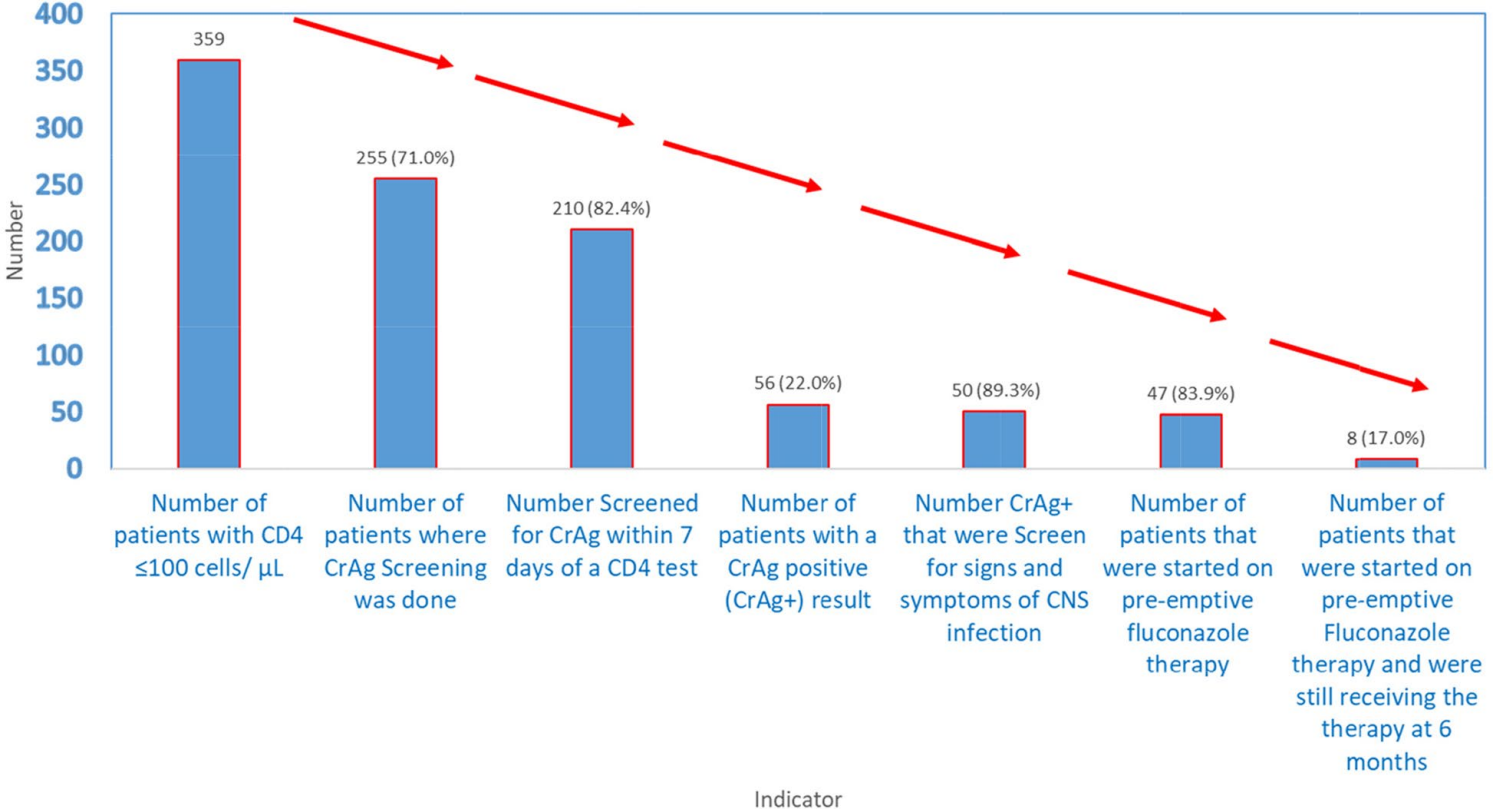
The number of patients in the study sample at each stage of the CrAg Screening Cascade (*n* = 359)

Study personnel, together with the EMR/ CD4 register focal person, generated a list of patient IDs with baseline CD4 ≤ 100 and tracked these in the CrAg/laboratory register to ascertain whether or not they underwent CrAg screening. When information was not available or missing from the CrAg/laboratory register, patient charts were retrieved by peer patients/ expert clients (*these are patients with HIV who have freely disclosed their HIV status and perform low level tasks at HIV clinics including treatment education, file retrieval and taking patients’ vitals*), and the study team abstracted data related to the CrAg screening program. Figure 1 illustrates the number of patients at each stage of the CrAg screening cascade.

#### Part 2: interviews with health facility staff

We developed a questionnaire to identify the key barriers and facilitators of a successful CrAg screening cascade and piloted it among staff at the IDI-Mulago ART clinic. We believed that such a questionnaire would seek important information related to the day-to-day running of the CrAg screening program direct from the implementers, and further explain the findings of the register/chart reviews. Information collected by this questionnaire were organised under the following themes: 1) health facility’s capacity to do CD4 testing for all newly diagnosed HIV patients, 2) staff training on CrAg screening since Jan 2016, 3) stocks of CrAg screening kits and the mechanism for replenishment, 4) capacity (availability of human resource and equipment) of the health facility to do lumbar punctures and 5) availability and prescriptions of fluconazole.

On the same day of the CrAg register/chart review or the next day, the study team located the laboratory personnel and ART in-charge and administered the interviews after obtaining their consent. All the data from the registers and interviews were directly entered into a REDCap® [22] database administered on a tablet.

### Data management and analysis

We developed data abstraction tools that included variables reflecting the different steps along the CrAg screening cascade. The study tools were pre-tested at the IDI HIV clinic within Mulago National Referral Hospital in Kampala, Uganda. We then designed a database and survey using REDCap electronic data capture, hosted by the University of Minnesota [22, 23]. We included data validation rules within the data capture forms, and data were collected in both online and offline modes. One investigator managed the REDCap database and would inform the data collection team of any errors for immediate correction on a daily basis. A cleaned data set was exported to Stata version 14 (College Station, Texas) [24] for analysis. We summarized baseline CD4 using medians and interquartile ranges (IQR) and categorical variables were summarized using percentages. To test for the equality of proportions and medians between regions, we performed chi-square proportion tests for categorical variables, and Mann Whitney tests for median. We presented the overall proportion of patients that completed each step of the cascade using a table and bar graph.

## Results

### Chart audit/review findings

Our analysis included 359 (76.1%) out of the expected 472 patient charts. All but one health facility were government health facilities, and the majority (135/359, 37.6%) were health centres at level III (Table 1).

**Table 1:** Background characteristics of patients in the study sample, stratified by urban/rural status of the health facility where their record was reviewed and overall (*n* = 359)

		Location of site					
Characteristic (s)	Categories	Rural		Urban		Overall	
		N	%	N	%	N	%
Ownership of Health Facility	Government	149	99.3	209	100.0	358	99.7
	Private	1	0.7			1	0.3
Health Facility Level	HC III	14	9.3	121	57.9	135	37.6
	HC IV	25	16.7	18	8.6	43	12.0
	General Hospital	29	19.3	40	19.1	69	19.2
	Referral Hospital	82	54.7	30	14.4	112	31.2
Baseline CD4 cell count	Median (IQR)	49 (25–71)		44 (19–70)		45 (21–71)	
	< 50	77	51.3	116	55.5	193	53.8
	50–100	73	48.7	93	44.5	166	46.2
Baseline WHO clinical stage	1	20	13.3	45	21.5	65	18.1
	2	52	34.7	63	30.1	115	32.0
	3	42	28.0	47	22.5	89	24.8
	4	35	23.3	54	25.8	89	24.8
	Missing	1	0.7			1	0.3

Of the 359 patients, the overall median (IQR) CD4 cell count was 45 cells/μL (21–71), 193 (53.8%) patients had a baseline CD4 cell count of < 50 cells/μL and 178/359 (49.5%) patients were classified with WHO clinical stage 3 or 4 HIV disease. As illustrated in Tables 2, 255/359 (71.0% (95% CI, 66.0–75.7) of the patients were CrAg screened and the proportion was higher among urban facilities compared to rural facilities (81.3% vs 56.7% *P* < 0.001). Ninety four percent (210/255) of the CrAg tests were done within 7 days of receiving a CD4 count result. Twenty two percent (56/255, 95% CI, 17.0–27.5%) of patients screened were CrAg positive (had cryptococcal antigenemia). Of the CrAg positive patients, 50/56 (89.3, 95% CI, 78.1–96.0) presented with at least one CNS sign or symptom and 39/56 (69.6, 95% CI, 55.9–81.2) had a lumbar puncture performed. While 39/48 (81.3% (95% CI, 67.4–91.1) of patients with CNS symptoms in urban health facilities had diagnostic lumbar punctures done, none was done in any of the rural health facilities. In patients with confirmed CrAg antigenemia, 47/56 (83.9, 95% CI, 71.7–92.4) were initiated on fluconazolepre-emptive therapy. At 6 months follow up, 8/47 (17.0, 95% CI, 7.6–30.8%) were still receiving fluconazole pre-emptive therapy. Among patients who had started ART, nearly 40% (129/332, 38.9%) started ART prior to having a CrAg test.

**Table 2:** The number of patients in the study sample at each stage of the CrAg Screening Cascade, stratified by urban/rural status of the health facility where their record was reviewed and overall (*n* = 359)

Stage on the CrAg cascade	Location of site			*P*-value
	Urban	Rural	Overall	
	Number: Proportion % (95% CI)	Number: Proportion % (95% CI)	Number: Proportion % (95% CI)	
Patients with CD4 ≤ 100 μ/ml	209 (58.2)	150 (41.8)	359 (100.0)	
Patients with CrAg screen	170: 81.3 (75.4–86.4)	85: 56.7 (48.3–64.7)	255: 71.0 (66.0–75.7)	< 0.001
Patients screened for CrAg within 7 days of a CD4 test	138: 81.2 (74.5–86.8)	72: 84.7 (75.3–91.6)	210: 82.4 (53.4–63.8)	0.489
Patients with CrAg screen contacted with results	170: 100.0 (97.9–100.0)	82: 96.5 (90.0–99.3)	252: 98.8 (96.6–99.8)	0.014
Patients with a CrAg positive result	48: 28.2 (21.5–35.0)	8: 9.4 (4.2–17.7)	56: 22.0 (17.0–27.5)	0.001
Patients screen for signs and symptoms of CNS infection	43: 89.6 (77.3–96.5)	7: 87.5 (47.3–99.7)	50: 89.3 (78.1–96.0)	0.860
Patients in whom a lumbar puncture was done	39: 81.3 (67.4–91.1)	0: 0 (0.0–0.0)	39: 69.6 (55.9–81.2)	
Patients started on pre-emptive fluconazole therapy	39: 81.3 (67.4–91.1)	8: 100.0 (63.1–100.0)	47: 83.9 (71.7–92.4)	0.181
Patients started on fluconazole that were still receiving pre-emptive fluconazole at 6 months	5: 12.8 (4.5–28.8)	3: 37.5 (8.5–75.5)	8: 17.0 (7.6–30.8)	0.108
Patients who were started on ART	183: 87.6 (82.3–91.7)	149: 99.3 (96.3–100.0)	332: 92.5 (89.2–95.0)	< 0.001
Patients who were started on ART prior to CrAg screening	130: 71.0 (63.9–77.5)	53: 35.6 (27.9–43.8)	129: 38.9 (33.6–44.3)	< 0.001
Patients with a CD4 ≤ 100 μ/ml that were reportedly alive at the time of data collection	110: 52.6 (45.6–59.6)	103: 68.7 (60.6–76.0)	213: 59.3 (54.1–64.5)	0.002

### Barriers for a successful CrAg screening program

Results of semi-structured interviews of the laboratory personnel and ART clinic in-charges are shown in Table 3 where 58.0% of the respondents reported lack of sufficient equipment and supplies for the CrAg screening process while 50% indicated training gaps in CrAg screening, cryptococcal meningitis management, and lumbar puncture procedures as the main barriers to implementing a successful CrAg screening program.

**Table 3:** Areas for improvement in the facility operations of the CrAg Screening Cascade, as cited by laboratory personnel and ART clinic in-charges at facilities in the study sample (*n* = 26)

Barrier(s) cited	Respondent		
	Laboratory Personnel (*n* = 13)	ART clinic In-charge (*n* = 13)	All respondents (*n* = 26)
Lack of Equipment and Supplies	8 (62%)	7 (54%)	15 (58%)
Inadequate Human resources	2 (15%)	2 (15%)	4 (15%)
Training Gap (Need for a training)	5 (38%)	8 (62%)	13 (50%)
Do not have Guidelines and Policies	1 (8%)	1 (8%)	2 (8%)
Logistics	0 (0%)	2 (15%)	2 (8%)

## Discussion

In this study, we observed successes as well as critical gaps along the CrAg screening cascade in patients with lower CD4 cell count. Among eligible patients for CrAg screening, seven out of every ten were subsequently screened, over 80% of eligible patients was initiated on fluconazole, pre-emptive therapy but less than one in five of these were still receiving fluconazole after 6 months, in contrast to the Uganda Ministry of Health guidelines, which recommend 6 months of fluconazole pre-emptive therapy for cryptococcal antigenemia. Lack of supplies and training gaps were identified by health workers as impediments to a successful CrAg screening program.

Knowing the estimates of opportunistic infections among people living with HIV is very crucial in designing prevention strategies and major treatment needs [3], this has been fostered through strategically choosing the order and type of tests to include in the diagnostic algorithms for better care delivery. However, these algorithms need continuous evaluation to identify critical areas for improvement. Cryptococcal antigen screening is a major screening tool among patients with advanced HIV disease aimed at preventing morbidity and mortality due to cryptococcal infection. Integration of routine CrAg screening and pre-emptive fluconazole therapy in HIV care programs is cost effective with reduction of cryptococcal meningitis and overall reduction in HIV associated mortality.

Our evaluation study found higher CrAg screening in the urban facilities (81.3%) compared to rural facilities (56.7%), this may be associated with decreased logistical supplies in the rural areas, In addition, current WHO guideline recommendations of the ART test and treat policy in resource limited settings with decreased access to CD4 machines so as to prevent further delay in ART initiation, which is so profound in the rural areas.

We found a cryptococcal antigenemia prevalence of 22%. This was higher as compared to earlier analyses by Rajasingham et al, which found a global cryptococcal prevalence of 6% (95% CI, 5.8–6.2) [25]. In addition a study by Meya et al that assessed the cost effectiveness of serum CrAg screening in patients with HIV initiating ART with CD4 < 100 in resource limited settings in Uganda had a cryptococcal antigenemia prevalence of 8.2% [12]. There has not been a clear reason for such a higher prevalence detected, however this might be associated with the increased roll out of an organised CrAg screening program in the different areas of the country as compared before, with more capture of CrAg screening data, and increased number of ART experienced patients presenting with advanced disease. This could act as an eye opener for epidemiologists and policymakers that the burden of disease might be higher than the current estimates and thus a more thorough evaluation for better channelling of services. A higher percentage of CrAg positive patients had at least one central nervous system (CNS) sign and symptom suggesting a diagnosis of meningitis, which might be explained by the delayed presentation at the facility with advancement of the disease or symptomatic antigenemia and some presenting with subclinical meningitis. Unlike previous studies, a big percentage of patients were ART experienced prior to the CrAg screening with some patients failing on their regimens, recently adherent and others having initiated ART a few weeks prior to screening, exposing this category of patients to a higher risk of developing unmasking cryptococcal meningitis immune reconstitution inflammatory syndrome (CM IRIS). In the study by Meya et al, all five of the patients with cryptococcal antigenemia who were initiated on ART but not treated with fluconazole died [12]. Therefore, we propose that these patients have a higher chance of dying despite reaching facilities with better care. Patients screened in the urban centres were more likely to have a diagnostic lumbar puncture as compared to rural facilities. This can be associated to increased availability of specialized and trained health personnel, logistical supplies, and reduced stigma towards lumbar punctures due to improved education status in urban facilities. In addition, a lot of patients who require services like this are referred from village facilities to urban facilities, as described in the three delay model of maternal health seeking [26], some patients end up not making it to the facilities and first seek alternative care like herbal medicine or traditional healers. Some who do so may delay, thus presenting later on with very advanced disease.

Despite the fact that a big percentage of patients were initiated on pre-emptive fluconazole after having a positive CrAg, which could be related to the fact that fluconazole is provided free of cost at most of government ART care centres in Uganda, the 6 month follow up was significantly low with only 17.0% still in care and on fluconazole. As demonstrated in number of studies, clinical cryptococcal meningitis develops in a certain number of patients even with pre-emptive fluconazole therapy. A study by Wake et al also showed that patients with cryptococcal antigenemia had 3.3 times increased risk of dying as compared to CrAg negative, and this remained significant even when adjusted for baseline CD4 (< 50 cells/μL) [27]. Further, these are patients with severe immunosuppression making them prone to other opportunistic infections before their CD4 counts improve. Therefore, follow up of these patients after initiating fluconazole pre-emptive therapy is critical and ignoring this gap might undermine all the efforts established to reduce cryptococcal meningitis in Uganda and similar settings in Sub-Saharan Africa.

### Study limitations

We were unable to locate and review some of the patients’ files and thus our findings are based on a reduced (76.1% of expected patients’ records) sample size. Such lost files were for patients classified as dead at the clinic or lost to follow up, and this arose out of a failure or complete absence of streamlined filing system at the health facility. The failure to review such patients’ files could have resulted into a possibility of under estimating the metrics along the CrAg screening cascade as presented here, this may be avoided in future studies by accounting for higher percentage of samples with missing data in the initial sample size estimation. Secondly, like any other retrospective study, we encountered a number of files with missing data despite the fact that such patients had met the inclusion criteria. Others were withdrawn from the analysis because of poor documentation that made ascertainment of metrics along the CrAg cascade difficult; and this further impacted on our sample size. The passive nature of surveillance in almost all health facilities made it impossible for us to quantify all the patients that had died during the 6 months follow up as some had passed away in the community with no data captured in the patient chart at the facility. Due to lack of a centralised system for HIV care and CrAg screening, there is a possibility that some of the patients referred from lower level facilities to higher level facilities could be counted twice resulting in overestimation of metrics in the CrAg screening care cascade Facilities were selected via a convenience sample, which makes the generalizability of these results limited.

## Conclusions

There is a critical gap in follow up of patients after they initiate pre-emptive fluconazole therapy. A high proportion of patients were ART experienced and presented with advanced HIV disease, thus exposing them to an increased risk of unmasking cryptococcal meningitis. There is great need for logistical support, test kits and refresher training of health worker personnel on CrAg screening, cryptococcal meningitis management, and lumbar puncture procedures especially in rural facilities. A more thorough evaluation to identify gaps and successes of the CrAg screening program is /warranted.

## Data Availability

The datasets used or analysed during the current study are available from the corresponding author on reasonable request.
